# Genome-wide association study identifies *SIAH3* locus influencing the rate of ventricular enlargement in non-demented elders

**DOI:** 10.18632/aging.102435

**Published:** 2019-11-11

**Authors:** Xian Li, Shu-Guang Chu, Xue-Ning Shen, Xiao-He Hou, Wei Xu, Ya-Nan Ou, Qiang Dong, Lan Tan, Jin-Tai Yu

**Affiliations:** 1 Department of Neurology, Qingdao Municipal Hospital, Dalian Medical University, Dalian, China; 2 Department of Radiology, Shanghai East Hospital, Tongji University School of Medicine, Shanghai, China; 3 Department of Neurology and Institute of Neurology, Huashan Hospital, Shanghai Medical College, Fudan University, Shanghai, China; 4 Department of Neurology, Qingdao Municipal Hospital, Qingdao University, Qingdao, China

**Keywords:** genome-wide association study, ventricular enlargement, neurodegenerative diseases, SIAH3

## Abstract

Ventricular enlargement occurs in several neurodegenerative and psychiatric diseases. A large genome-wide association study (GWAS) has identified seven loci associated with ventricular volume. The rate of ventricular enlargement increased in the progression of disease from normal cognition to dementia. Here, we aimed to use the rate of ventricular enlargement as an endophenotype for the development and progression of neurodegenerative diseases to discover more common genetic variants. We performed a GWAS of the rate of ventricular enlargement using 507 nondemented non-Hispanic white participants from the Alzheimer’s Disease Neuroimaging Initiative (ADNI) cohort. Linear regression model was used to identify the association of the rate of ventricular enlargement with single nucleotide polymorphisms (SNPs) in PLINK software. The associations of genome-wide significant SNPs with other four phenotypes were further discussed. Two SNPs (rs11620312, P = 4.04×10^−8^; rs79174114, P = 4.28×10^−8^) within *SIAH3* gene in linkage disequilibrium (LD) reached genome-wide significance for association with increased rate of ventricular enlargement. Some intergenic SNPs and SNPs within *NKAIN2, TBC1D2, GALNT18, ABCC1* and *SRCIN1* genes were identified as potential candidates. *SIAH3* rs11620312-C carriers were associated with poor cognition and brain hypometabolism longitudinally. Our findings indicated that *SIAH3* gene may have potential influence on the pathogenesis of neurodegenerative diseases.

## INTRODUCTION

Ventricular enlargement occurs in normal elders, patients with some neurodegenerative diseases, such as Alzheimer disease (AD) [1] and Parkinson’s disease (PD) [2], and patients with some psychiatric diseases, such as schizophrenia [3]. The rate of ventricular enlargement was suggested to be a sensitive marker of AD progression [4–6]. And it was increased in the progression from normal cognition to dementia [7]. Moreover, it was increased in PD with mild cognitive impairment (MCI), and it was suggested as a potential marker for PD [8]. Although ventricular enlargement was usually thought as a symbol of brain atrophy, a few studies showed that it was independently heritable [9, 10]. Recently, a large genome-wide association study (GWAS) identified seven genetic loci associated with ventricular volume [11], but no GWAS focused on genetic risk loci associated with the rate of ventricular enlargement.

The GWAS method has been used to discover genetic risk factors. Traditional case-control based GWAS may be confounded by preclinical status before onset of diseases, and the studies focused on genes associated with disease risk rather than with other aspects of diseases such as progression or biomarkers [12]. Endophenotypes are quantitative traits strongly associated with diseases that also share genetic architecture with diseases. Endophenotype-based GWAS can increase statistical power and avoid the limitations of case-control based GWAS [13, 14].

We hypothesized that some genetic loci may be involved in the pathogenesis of neurodegenerative diseases. Thus, to test this hypothesis and based on the role of the rate of ventricular enlargement in neurodegenerative diseases, we conducted a GWAS using the rate of ventricular enlargement as an endophenotype in Alzheimer’s Disease Neuroimaging Initiative (ADNI) cohort to explore more genetic risk loci.

## RESULTS

### Demographics and the rate of ventricular enlargement

In this study, a total of 507 nondemented non-Hispanic white participants [cognitively normal (CN) = 211, MCI = 339] whose data satisfied all quality control criteria from the ADNI cohort were included after quality control procedures. The details of demographic information and the endophenotype have been shown in Table 1.

**Table 1:** Demographics and the endophenotype for the GWAS samples.

	**CN**	**MCI**	**Total**
Sample size, n	196	311	507
Age, mean (SD), y	74.7(5.3)	72.0(7.3)	73.0(6.7)
F, n (%)	99 (51.0)	115 (37.0)	214 (42.2)
*APOE* ε4 carrier, %	26.5	46.3	38.7
Ventricular enlargement rate, mean (SD)	4.9×10^−2^ (3.1×10^−2^)	6.5×10^−2^ (4.9×10^−2^)	5.9×10^−2^ (4.4×10^−2^)

Abbreviations: GWAS: genome-wide association study; CN: cognitively normal; MCI: mild cognitive impairment; SD: standard deviation; F: female.

### GWAS results

**Figure 1:**
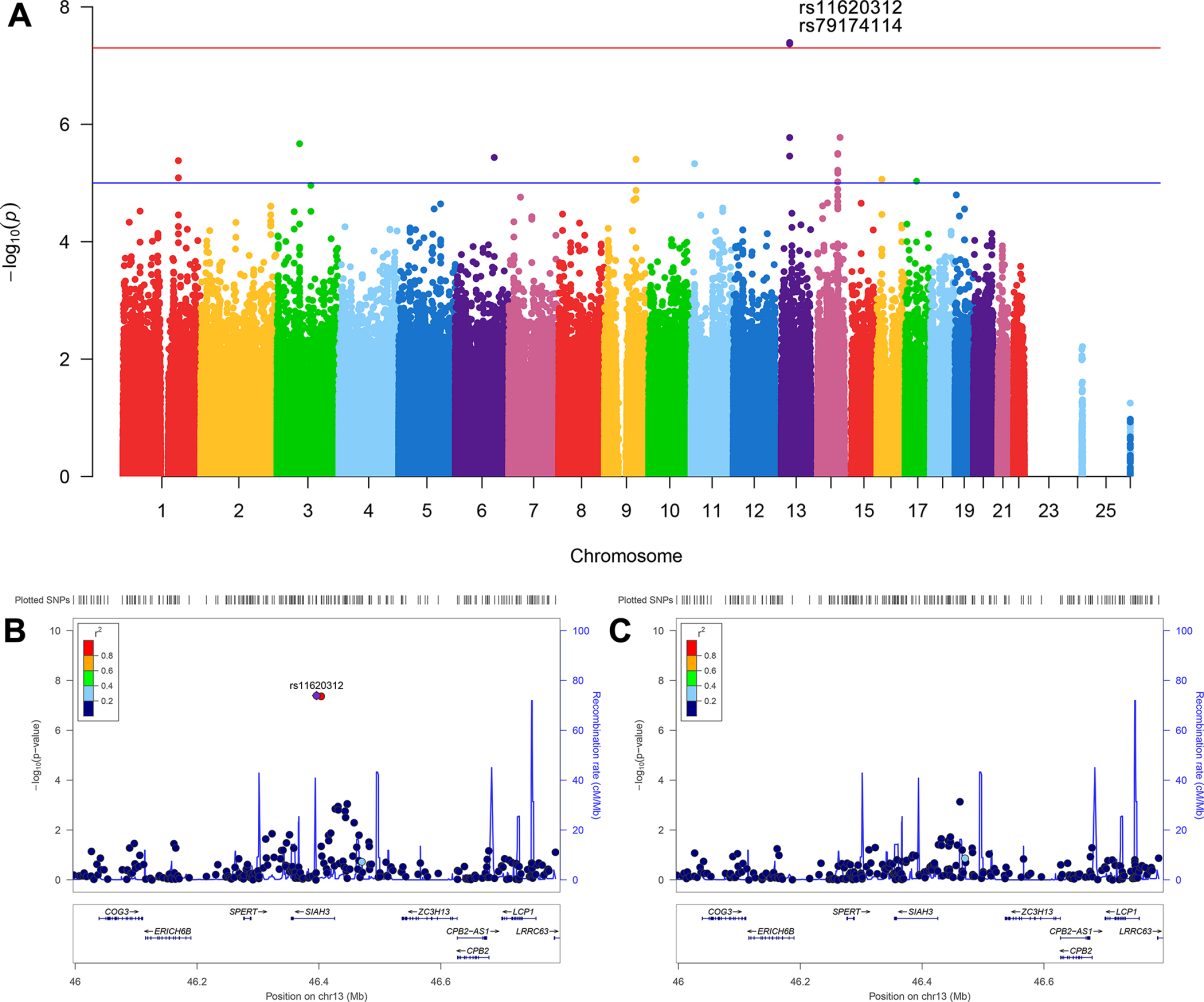
**Manhattan plot and regional association plots.** (**A**) Manhattan plot for association with the rate of ventricular enlargement, after adjusting for age, gender, *APOE* ε4, ICV, MRI scanner type and the first three principal components. The red line is the genome-wide significant threshold at P = 5×10^-8^; the blue line is a suggestive threshold at P = 10^-5^. (**B**) Regional association plot for rs11620312 in the *SIAH3* gene on chromosome 13. (**C**) Regional association plot for the *SIAH3* gene after controlling for rs11620312. No SNPs showed significant association after controlling for rs11620312, suggesting the associations were driven by rs11620312. Abbreviation: ICV = intracranial volume; MRI = magnetic resonance imaging; P = P value.

**Figure 2:**
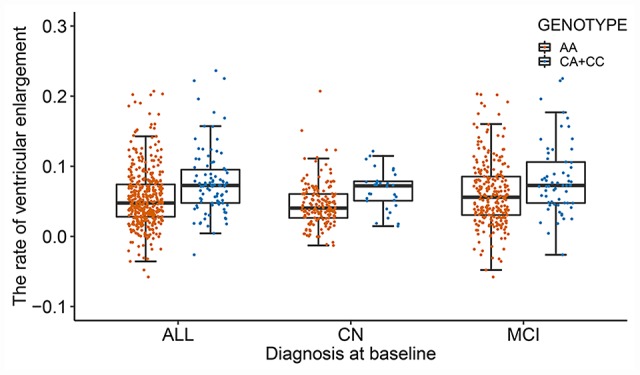
**The differences in the rate of ventricular enlargement between the two genotypes in total subjects and each diagnostic group.** The minor allele (C) of rs11620312 carriers had increased rates of ventricular enlargement in all subjects (P = 3.26×10^-7^), CN group (P =1.23×10^-4^) and MCI group (P = 9.08×10^-4^). Abbreviations: CN = cognitively normal; MCI = mild cognitive impairment; P = P value.

After adjusting for age, gender, apolipoprotein E (*APOE*) ε4, total intracranial volume (ICV), magnetic resonance imaging (MRI) scanner type (1.5T versus 3T) at baseline and the first three principal components (PCs), the effect of population stratification was well controlled for (genomic inflation factor λ= 1.00, Supplementary Figure 2). Two single nucleotide polymorphisms (SNPs) (rs11620312, P = 4.04×10^-8^; rs79174114, P = 4.28×10^-8^) on chromosome 13 were found to have genome-wide significant associations with the rate of ventricular enlargement (Figure 1A and Table 2). Rs11620312 and rs79174114 within the *SIAH3* gene are in linkage disequilibrium (LD, r^2^ = 0.96, D’ = 0.99) (Figure 1B), and after controlling for the rs11620312 genotype, no SNPs showed strong association with the rate of ventricular enlargement (Figure 1C). we regard the rs11620312 was index SNP. Carriers of the minor allele (C) of rs11620312 had increased rates of ventricular enlargement in all subjects (P = 3.26×10^-7^), CN group (P =1.23×10^-4^) and MCI group (P = 9.08×10^-4^) (Figure 2). Although there is no statistical significance, the minor allele (C) of rs11620312 and the minor allele (T) of rs79174114 may be associated with increased trend of *SIAH3* expression in brain tissues according to preliminary data from the UKBEC database (Supplementary Figure 3). Suggestive associations of several SNPs with the rate of ventricular enlargement (P < 10^-5^) were also detected (Figure 1A and Table 2), including two other SNPs in *SIAH3*, eight intergenic SNPs and six SNPs within *NKAIN2, TBC1D2, GALNT18, ABCC1, SRCIN1* genes.

**Table 2:** Genome-wide significant and suggestive SNPs associated with the rate of ventricular enlargement.

**CHR**	**SNP**	**MA(MAF)**	**GENE**	**SNP type**	**β**	**P**
13	rs11620312	C (0.12)	*SIAH3*	Intron	0.023	4.04×10^-8^
13	rs79174114	T (0.11)	*SIAH3*	Intron	0.023	4.28×10^-8^
1	rs1885646	A (0.15)	*--*	Intergenic	0.017	4.16×10^-6^
3	rs9821691	G (0.43)	*--*	Intergenic	0.013	2.14×10^-6^
6	rs2626129	C (0.32)	*NKAIN2*	Intron	-0.015	3.67×10^-6^
9	rs10985425	G (0.05)	*TBC1D2*	Intron	0.018	3.95×10^-6^
11	rs1994399	G (0.39)	*GALNT18*	Intron	0.012	4.69×10^-6^
13	rs11618124	T (0.33)	*SIAH3*	Intron	0.016	1.68×10^-6^
13	rs1998892	C (0.37)	*SIAH3*	Intron	0.017	3.48×10^-6^
14	rs8022233	T (0.42)	*--*	Intergenic	0.012	9.57×10^-6^
14	rs12434273	C (0.40)	*--*	Intergenic	0.012	6.06×10^-6^
14	rs12894449	G (0.48)	*--*	Intergenic	0.013	3.24×10^-6^
14	rs2998298	A (0.47)	*--*	Intergenic	0.013	3.14×10^-6^
14	rs2922629	T (0.30)	*--*	Intergenic	0.012	6.80×10^-6^
14	rs67783323	G (0.15)	*--*	Intergenic	0.018	1.67×10^-6^
16	rs4781701	C (0.13)	*ABCC1*	Intron	0.015	8.67×10^-6^
16	rs12922404	T (0.12)	*ABCC1*	Intron	0.015	8.67×10^-6^
17	rs2075051	T (0.32)	*SRCIN1*	Intron	0.012	9.32×10^-6^

Note: Two significant SNPs (rs11620312 and rs79174114) were in linkage disequilibrium (r^2^>0.8). Abbreviation: CHR = chromosome; SNP = single nucleotide polymorphism; MA = minor allele; MAF = minor allele frequency; β = standardized effect size; P = p-value.

### Association between rs11620312 and other phenotypes

**Figure 3:**
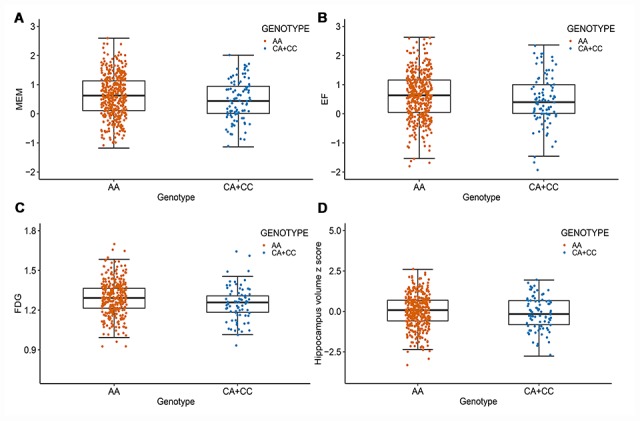
**Rs11620312 associated with other phenotypes at baseline.** The minor allele (C) of rs11620312 was not associated with MEM (**A**, P_Bonf_ = 0.29), EF (**B**, P_Bonf_ = 0.32), FDG (**C**, P_Bonf_ = 0.06) and hippocampus volume (**D**, P_Bonf_ = 0.26) at baseline. Abbreviation: MEM = cognitive score for memory; EF = cognitive score for executive functioning; FDG = 18F-fluorodeoxyglucose.

**Figure 4:**
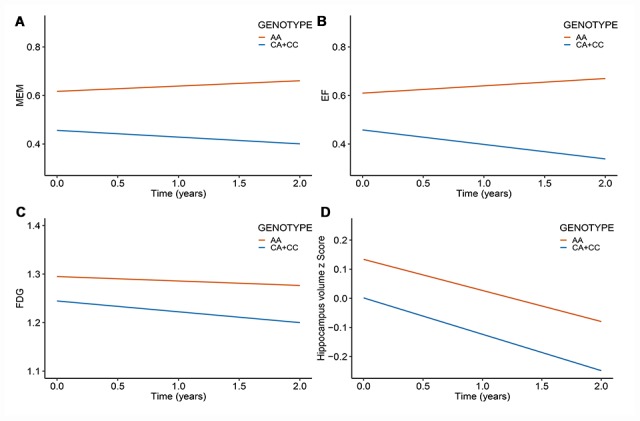
**Rs11620312 associated with other phenotypes longitudinally.** The minor allele (C) of rs11620312 was associated with the increased rates of EF decline (**B**, P_Bonf_ = 0.037) and FDG hypometabolism (**C**, P_Bonf_ = 0.029), however it was not associated with the change rates of MEM (**A**, P_Bonf_ = 0.18) and hippocampus volume (**D**, P_Bonf_ = 1.16) over time. Abbreviation: MEM = cognitive score for memory; EF = cognitive score for executive functioning; FDG = 18F-fluorodeoxyglucose.

After correcting for multiple comparisons using the Bonferroni procedure, the minor allele (C) of rs11620312 was not associated with memory (MEM) (P_Bonf_ = 0.29), executive functioning (EF) (P_Bonf_ = 0.32), 18F-fluorodeoxyglucose (FDG) metabolism (P_Bonf_ = 0.06) and hippocampus volume (P_Bonf_ = 0.26) at baseline (Figure 3). However, it was correlated with accelerated rates of decline in EF (P_Bonf_ = 0.037) and FDG (P_Bonf_ = 0.029) within 2 years (Figure 4), suggesting that the minor allele (C) of rs11620312 may be associated with accelerated cognitive decline and brain hypometabolism over time.

## DISCUSSION

We identified two novel genome-wide significant SNPs in LD (rs11620312 and rs79174114, r^2^ > 0.8) within *SIAH3* gene and sixteen other suggestive loci (in *NKAIN2, TBC1D2, GALNT18, ABCC1, SRCIN1* genes and intergenic region). Moreover, we found the minor allele (C) of rs11620312 may be associated with accelerated cognitive decline and brain hypometabolism over time. Our findings suggest that *SIAH3* gene, as a novel genetic factor, may be involved in the pathogenesis of neurodegenerative diseases.

*SIAH3* (siah E3 ubiquitin protein ligase family member 3) gene is located on chromosome 13, encoding a member of the seven in absentia (Sina) protein family [15]. Although its function was not completely clear, there were some reports found that the SIAH3 gene played a role in high Cd placentas [16], metastatic prostate tumors [15] and human gingiva following surgical wounding [17]. A study found that SIAH3 was localized to mitochondria and it could inhibit PINK1 (PTEN-induced putative kinase 1) accumulation as a negative regulator after mitochondrial injury [18]. PINK1 plays an important role in mitochondrial autophagic pathway (mitophagy) by accumulating on the surface of the damaged mitochondrial outer membrane, as well as subsequently recruiting and activating Parkin [19]. It has been confirmed that PINK1/Parkin mitophagy was involved in the pathogenesis of PD [20]. Moreover, it was associated with other neurodegenerative diseases related to mitochondrial dysfunction, such as AD and multiple sclerosis (MS) [21–23].

Mitochondrial dysfunction, reducing intracellular adenosine triphosphate (ATP) levels and increasing reactive oxygen species (ROS) production, has been identified as an important mechanism in multiple neurodegenerative diseases [24]. It can lead to abnormal accumulation of Aβ and tau which are involved in the pathogenesis and pathology of AD [25]. PINK1/Parkin mitophagy is a protective pathway that can eliminate the severely damaged mitochondria to reduce toxic products and provide enough energy [26]. Moreover, according to a recent study, decreased expression of PINK1 is associated with increased Aβ accumulation, mitochondrial dysfunction, and impairments in learning and memory in a mouse model of Alzheimer disease [23]. In our study, we found two genome-wide significant SNPs (rs11620312 and rs79174114) within the *SIAH3* gene were associated with an increased rate of ventricular enlargement in 507 nondemented elderly individuals. Thus, we hypothesized that SIAH3 can affect the PINK1/Parkin mitophagy by inhibiting the accumulation of PINK1 in the damaged mitochondria, subsequently leading to mitochondrial dysfunction involved in the pathology and pathogenesis of neurodegenerative diseases.

In addition, we found sixteen suggestive SNPs which may have potential associations with neurodegenerative diseases, including two loci (rs11618124 and rs1998892) in *SIAH3*, eight intergenic SNPs and six SNPs in other genes, such as *NKAIN2* (rs2626129), *TBC1D2* (rs10985425), *GALNT18* (rs1994399), *ABCC1* (rs4781701, rs12922404), and *SRCIN1* (rs2075051). *NKAIN2* (Na^+^/K^+^ transporting ATPase interacting 2) gene, highly expressed in brain tissues, encodes one of transmembrane proteins that interact with β-subunits of Na^+^/K^+^-ATPase [27]. Although its function was not clear, a previous study suggested that it may have associations with neurologic phenotypes like severe psychomotor retardation associated with cerebral atrophy [28]. *TBC1D2* (TBC1 domain family member 2) encodes a GTPase-activating protein (GAP) for Rab7 GTPase, leading to Rab7 inactivation as well as the regulation of E-cadherin degradation and cell-cell adhesion [29]. The biological function of *GALNT18* (polypeptide N-acetylgalactosaminyltransferase 18) gene is unclear. *ABCC1* (ATP binding cassette subfamily C member 1) gene encodes a member of ATP-binding cassette (ABC) transporters involved in the multidrug resistance. A few studies found that *ABCC1* protein may be associated with Aβ accumulation in the brain [30, 31]. *SCRIN1* (SRC kinase signaling inhibitor 1) may be related with dendritic spine morphology and synaptic plasticity [32]. These genes, although not reaching the genome-wide significant level, may play a potential role in the pathogenesis of neurodegenerative diseases.

Our study has several limitations. First, the sample size of this study was moderate, which limited the statistical power of the GWAS and may give rise to false positive results. Moreover, the moderate sample size limited the statistical power of stratified analyses for each diagnostic group. Second, our sample was restricted to non-Hispanic white participants to avoid population stratification across ethnicities, but the rs11620312 in *SIAH3* has various frequencies in different races. The contradiction determines the racial limitation of our research and the necessity of replication analysis in other races. Third, post-GWAS analyses about *SIAH3* gene, like gene annotation and pathway analysis, were not available and the function of gene needs further exploration. Finally, more independent replication studies with large samples were needed to confirm these results.

In conclusion, we identified two novel loci (rs11620312 and rs79174114) within the *SIAH3* gene associated with an increased rate of ventricular enlargement. Our further study demonstrated that rs11620312 was related to poor cognition and brain hypometabolism over time. The biological function of *SIAH3* in mitochondrial dysfunction may have relevance for the pathogenesis of neurodegenerative diseases, which merits further investigation.

## MATERIALS AND METHODS

### Alzheimer’s Disease Neuroimaging Initiative (ADNI) database

Initial data used in this study were obtained from the ADNI database (http://adni.loni.usc.edu). ADNI database was launched in 2004, led by Principal Investigator Michael W. Weiner, MD. It’s a public, longitudinal and multicenter study to detect clinical, imaging, biochemical and genetic biomarkers of AD [33]. This database includes three cohorts, i.e. ADNI-1, ADNI-GO and ADNI-2. More details of the ADNI database were described in prior publications and on the website of the ADNI database (http://adni.loni.usc.edu/about/).

### Ethic

This study was approved by institutional review boards of all contributing research institutions, and informed consent in writing was acquired from all subjects or authorized agents.

### Subjects

The initial cohort included 550 nondemented participants with both data on ventricular volume at baseline and 2-year follow-up and genetic information from ADNI database. All participants were restricted to non-Hispanic white participants to reduce the confounding from population stratification in the GWAS. This step excluded 36 participants. Moreover, to detect the confounding from cryptic relatedness and population substructure, we did genomic identity-by-descent (IBD) and multidimensional scaling (MDS) analysis using the PLINK software [34] (Supplementary Figure 1). Four participants who clustered separately from the others were removed, resulting in 510 valid participants.

### Endophenotype and quality control

Measurements of ventricular volume in ADNI-1 and ADNI-GO/2 were performed on 1.5T and 3T MRI scanners, respectively, using T1-weighted sequences with the standard ADNI MRI protocols. More details about measurements of ventricular volume were described elsewhere [33, 35, 36] and on the ADNI website (http://adni.loni.usc.edu). The annualized percent change of ventricular volume at 2-year follow-up compared to baseline was used as endophenotype [37]. To reduce the potential for false positives, three extreme outliners (the rate of ventricular enlargement > mean ± four standard deviations) were excluded, resulting in 507 valid participants.

### Genotyping and quality control

Samples of ADNI-1 and ADNI-GO/2 cohorts were genotyped using the Illumina Human610-Quad, and HumanOmniExpress microarray chips (Illumina, Inc., San Diego, CA), respectively [14]. Quality control procedures were performed using PLINK software with the following criteria [38, 39]: call rate for SNPs >98%, call rate for individuals >95%, minor allele frequencies >0.05, and Hardy-Weinberg equilibrium test p > 0.001. A total of 1,231,747 SNPs were retained after cleaning. The polymorphisms rs7412 and rs429358, which define the *APOE* alleles, were genotyped separately by an *APOE* genotyping kit [40].

### Statistical analyses

GWAS was performed using linear regression under an additive genetic model (i.e., dose-dependent effect of the minor allele) in PLINK software. Age, gender, *APOE* ε4 [41], ICV and MRI scanner type (1.5T versus 3T) at baseline were included as covariates. Principal components analysis (PCA) was derived using genome-wide complex trait analysis (GCTA) software [42], and the first three PCs were included as covariates. Conservative thresholds of P values <5×10^-8^ and <10^-5^ were used to represent genome-wide significant and suggestive associations, respectively [43]. Manhattan plot and Quantile-Quantile (QQ) plot were drawn in R software (version 3.5.2), and regional association plots were visualized by Locus Zoom website [44]. Differences in the endophenotype (the rate of ventricular enlargement) between rs11620312-C carriers and non-carriers in all subjects and two diagnosis groups were examined using multiple linear regression adjusting for age, gender, *APOE* ε4, ICV and MRI scanner type (1.5T versus 3T) at baseline in R software. The effect of genome-wide significant SNPs on gene expression was detected by accessing data from the UKBEC (www.braineac.org) [45].

### Association between the genome-wide significant SNP and other phenotypes

We further detected the relationship between the genome-wide significant SNP and other phenotypes, including composite cognitive scores for EF [46] and MEM [47], FDG metabolism and hippocampus volume in the GWAS cohorts. These data were also obtained from the ADNI database (http://adni.loni.usc.edu). Statistical analyses used multiple linear regression models for cross-sectional studies and mixed-effect models for longitudinal studies in R software (version 3.5.2). Age, gender, and *APOE* ε4 were used as covariates for all phenotypes, and educational level and ICV were included in the models of cognitive scores and hippocampus, respectively. Adjusted P-values (P_Bonf_) were corrected for multiple comparisons using the Bonferroni procedure.

## Supplementary Material

Supplementary Figures
