# Supplementary material for: Genome-wide association study identifies SIAH3 locus influencing the rate of ventricular enlargement in non-demented elders
Source: Aging (Albany NY). 2019 Nov 11;11(21):9862–74. doi: 10.18632/aging.102435 (PMC6874439; doi:10.18632/aging.102435)
Supplement: Supplementary Figures [file aging-11-102435-s001.pdf]

SUPPLEMENTARY FIGURES

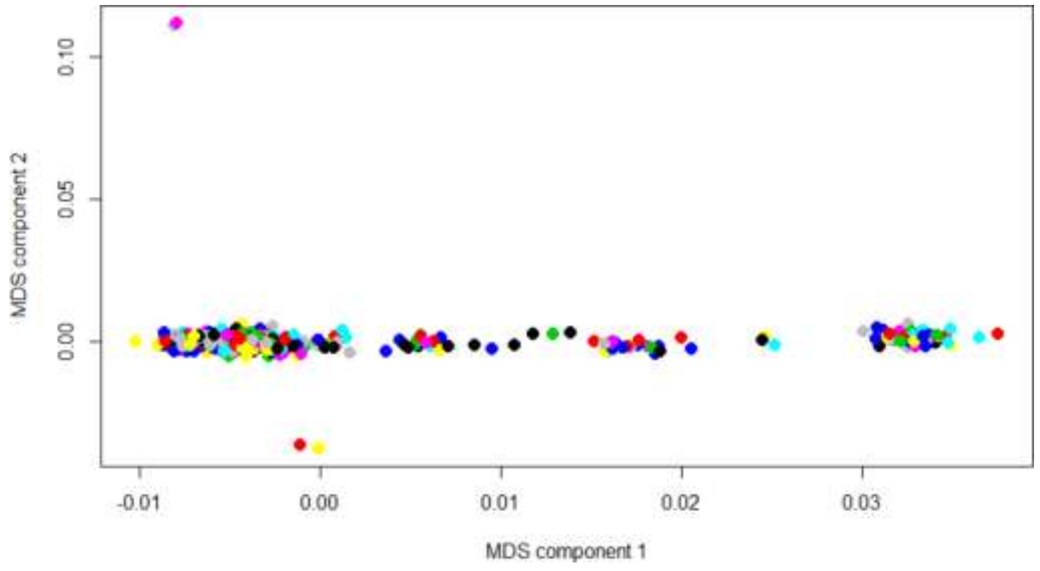

Supplementary Figure 1. Cryptic relatedness and population substructure were checked with genomic identity-by-descent (IBD) and multidimensional scaling (MDS) components. MDS plot of ADNI non-Hispanic Caucasian samples. Samples seemed to form loose clusters and four samples were outliers based on the second MDS component (at top of plot (137_S_4466 and 021_S_0159) and at bottom of plot (024_S_2239 and 024_S_4084)), suggesting potential population substructure. To check for cryptic relatedness, which can confound GWAS studies, pairwise identity-by-descent fraction (π) between each pair of samples were calculated using PLINK. Two related sample pair was identified (137_S_4466 and 021_S_0159, π = 0.50; 024_S_2239 and 024_S_4084, π = 0.42), which are probably first-degree relatives. No other cryptic relations were identified from the sample, at a threshold of π > 0.2. Abbreviation: GWAS = genome-wide association study.

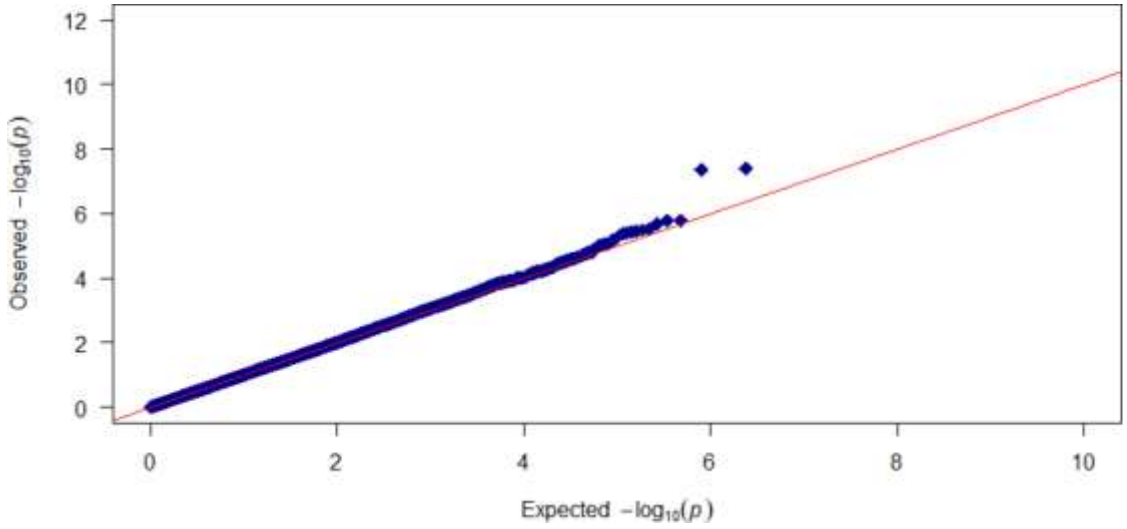

Supplementary Figure 2. The Quantile-Quantile plot of GWAS. Observed −log10 p-values (y-axis) were plotted against those expected p-values under the null hypothesis (x-axis). No substantial deviation from the red line was observed in the bulk of the distribution and the genomic inflation factors were λ = 1, indicating absence of population stratification or other confounding factors. Abbreviation: GWAS = genome-wide association study.

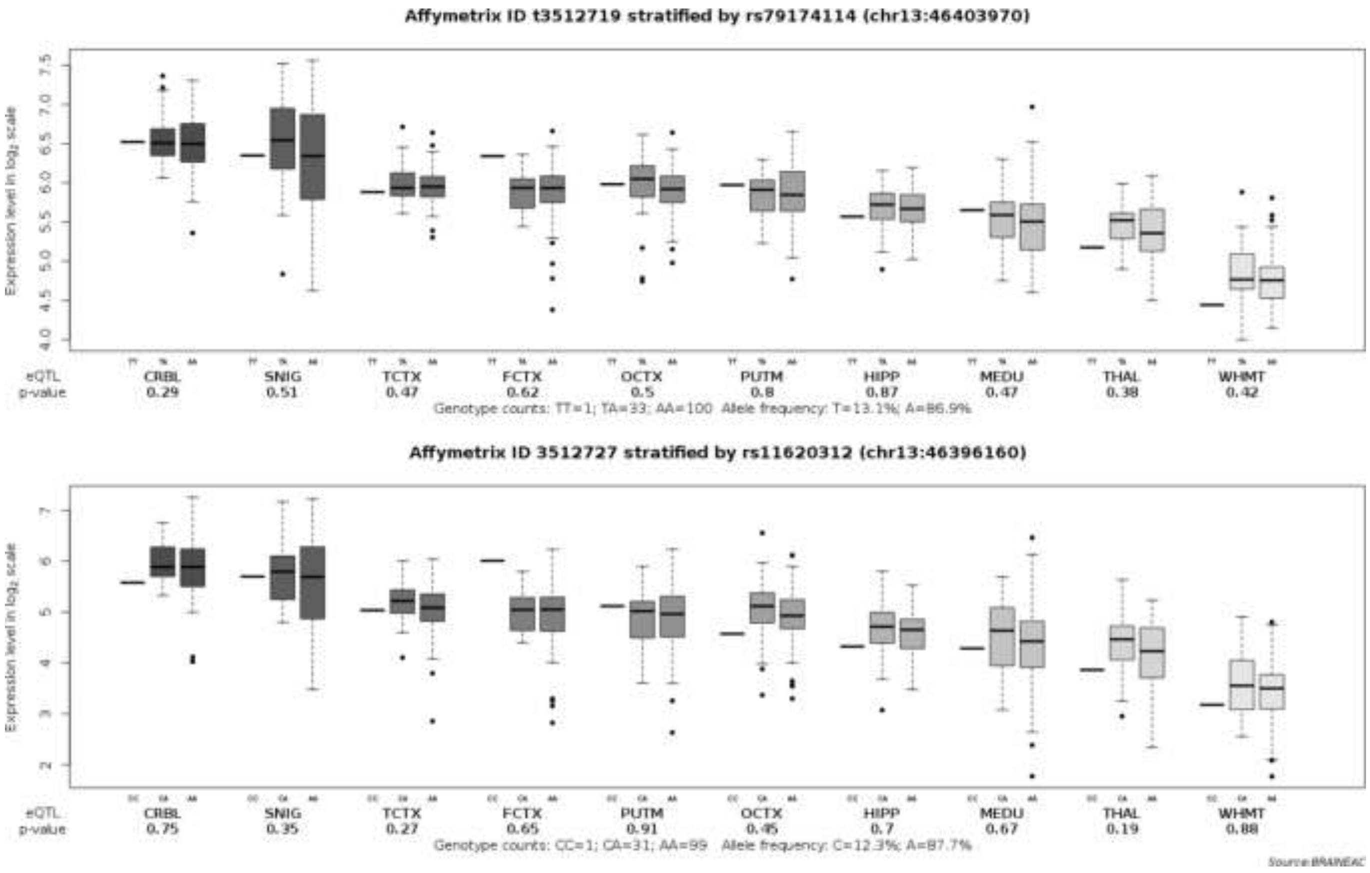

Supplementary Figure 3. Exon-specific expression level in ten brain tissues. Although there was no statistically significance, *SIAH3* gene was increased expression among the rs11620312-C carriers and rs79174114-T carriers in brain tissues according to preliminary data from the UKBEC database.
